# *‘They went for the test together but came back separately’*: a constructivist grounded theory perspective on male engagement in antenatal HIV testing in Bamenda, Cameroon

**DOI:** 10.1186/s12884-025-07134-w

**Published:** 2025-01-22

**Authors:** Lily Haritu Foglabenchi, Nancy Manigha Nyahkeh, Heidi Stöckl, Tanya Marchant

**Affiliations:** 1 AIDS Care and Prevention Program, Cameroon Baptist Convention Health Services, Bamenda, Cameroon; 2 Department of Disease Control, London School of Hygiene and Tropical Medicine, London, UK; 3 Ministry of Public Health (Njikwa Health District) Njikwa, Njikwa, Cameroon; 4 Institute for Medical Information Processing, Biometry and Epidemiology, Faculty of Medicine, Ludwig-Maximilians-Universität München, Munich, Germany

**Keywords:** Cameroon, Male involvement, HIV/AIDS, Gender, Grounded theory, Couple testing

## Abstract

**Introduction:**

Male engagement in HIV testing during pregnancy significantly contributes towards the prevention of maternal seroconversion and paediatric HIV acquisition. Despite this, men especially the male partners of pregnant women have been consistently missing in the HIV prevention cascade. The factors accounting for sub-optimal levels in male engagement intersect but reasons for this are poorly understood. Using the combined perspectives of pregnant women and their partners, this study aims to expand the evidence on the forces that influence prenatal HIV testing behaviours among couples in Bamenda, Cameroon.

**Methods:**

This qualitative study purposively selected pregnant women receiving prenatal care for semi-structured interviews (*n* = 38); focus group discussion (*n* = 6) and their male partners (*n* = 30 for semi-structured interviews and *n* = 6 for focus group discussion) in Nkwen Baptist Hospital—an urban hospital in Bamenda, Cameroon. Nvivo was used for data management and subsequently we performed a grounded theory analysis through memoing and constant comparisons.

**Results:**

Maternal HIV risk perception was the prominent theme intersecting with couple communication, perceptions on HIV testing outcome, and engagement of male partners by facility staff to influence couple prenatal HIV testing behaviours. Although participants recognised the need for couple HIV testing, individual, interspousal, structural and socio-cultural factors determined uptake of male partner testing. Perceptions on HIV risk were largely inaccurate and strongly gendered. For example, normative expectations on female fidelity were perceived as a buffer against HIV acquisition but this was not the norm regarding male partner behaviour. Also, couple communication was rare or subtle—mostly initiated by women who suspected spousal infidelity. For some men, HIV testing was a conscious decision to confirm fidelity, for others this was challenged by the fear of sero-discordant results and assumptions that maternal test results were a representation of their sero-status.

**Conclusion:**

Male partner involvement in prenatal HIV testing is largely influenced by gendered perceptions on HIV risk and couple testing outcomes. Given that these perceptions are moderated by spousal communication and the engagement of male partners by health facility staff, we call for gender-transformative interventions and policies that offer education on prenatal HIV risk, support couple communication and spousal disclosure.

**Supplementary Information:**

The online version contains supplementary material available at 10.1186/s12884-025-07134-w.

## Introduction

Male partners remain an overlooked population in the global HIV response despite being a key population that often determine HIV outcomes for women and children. In 2022, UNAIDS estimated that 1.3 million people became infected with HIV—far above the global target to reduce new HIV infections to below 370,000 annually by 2025 [1]. Evidence from studies in sub-Saharan Africa point to the fact that 60% to 94% of new HIV infections occur within married or co-habiting couples in heterosexual parternships [2–4]. Although women have increased susceptibility to HIV infection, men are disproportionately impacted once infected as they are missing in the HIV care cascade with approximately 50% of men less likely to know they are living with HIV and 27% less likely than women to access HIV treatment [5, 6]. Closing this gap is vital towards achieving the United Nations’ 95–95-95 target for epidemic control by 2030—whereby, 95% of people living with HIV are aware of their serostatus, of whom 95% are on lifesaving antiretroviral treatment (ART) and 95% are virally suppressed [7].

The World Health Organization (WHO) estimates that perinatal HIV transmission could range between 25% and 40% in the absence of any intervention [8]. Targeted intervention to identify women at risk of seroconversion through partner testing has been proposed as a key strategy in the elimination of maternal HIV transmission by 2030 [9]. While most countries mandate HIV testing for all pregnant women during their first antenatal visit and repeat testing with their male partners in subsequent visits, male engagement in developing countries has remained sub-optimal. Women have therefore continuously remained at risk of acquiring HIV from untested partners and this has been implicated in the growing number of HIV exposed infants globally [10].

Cameroon, a lower-middle-income country on the gulf of Guinea with a population of over 27 million has one of the highest HIV epidemics in West Africa and Central Africa [11, 12]. The country’s epidemic has been described as a concentrated epidemic within a generalised epidemic due to variations in the distribution of HIV within regions and sub-population groups [13]. Although the country is witnessing a progressive decline in its adult HIV prevalence rate from 5.4% in 2004 to 2.9% in 2022, glaring disparities have been noted between women and men [12, 14]. Women of reproductive age account for over half of adults living with HIV at a prevalence rate of 4.8%—over two times higher than their male counterparts at 2.0% [14]. A key contextual factor that contributes to high HIV incidence in the antenatal context in Cameroon and across sub-Saharan Africa is male sexual behaviours—whereby, relative to women, men are most likely to engage in multiple concurrent sexual partnerships and less likely to test for HIV during pregnancy [4, 15–18]. With respect to its PMTCT profile, Cameroon registered an increase in the mother-to-child transmission (MTCT) rate from 13.5% in 2015 to 16.8% in 2021 [12]. Furthermore, while the coverage of routine HIV testing among pregnant women was estimated at 90% in 2017, only 4.7% of their male partners reported to antenatal clinics for HIV testing [19–21].

Several studies have reported barriers to male involvement in antenatal HIV testing. These include but are not limited to: the fear of testing positive [22, 23], socio-cultural and gender norms [24, 25], a proxy claim on a pregnant partner’s negative status [23, 24] and the lack of time or conflicting work schedules with antenatal clinic hours [26–28]. These barriers occur in synergy and are under the influence of multiple realities [29, 30] yet, most studies and interventions employ an additive approach in addressing them while failing to account for the perspectives of both pregnant women and their male partners [31].

This study therefore sought to conceptually analyse the combined perspectives and experiences of pregnant couples to elucidate perceptions and behavioural norms that influence male engagement in prenatal HIV testing in Bamenda, Cameroon.

## Methods

This study was conducted in the context of a larger project exploring male involvement in HIV prevention, maternal, newborn and child health in Bamenda, Cameroon [32]. Field data collection took place between July and December 2021.

### Study design

This qualitative study integrated an interpretivist paradigm with the constructivist grounded theory approach through semi-structured interviews (SSIs) and focus group discussions (FGDs) [33, 34]. This approach was suitable for generating theoretical insights and a nuanced understanding on a social phenomenon like male HIV testing—given that it is underpinned by overlapping and multiple realities that need to be deconstructed to understand antenatal HIV testing behaviours and experiences.

### Study setting

Our study was conducted in the antenatal unit of Nkwen Baptist Hospital—a faith-based secondary health facility run by the Cameroon Baptist Convention Health Services [35]. The hospital serves a multi-ethnic population of about 350,000 residents and is centrally located in Bamenda—the regional capital of the North West Region of Cameroon. At the time of this study, the hospital had a well-established CDC/PEPFAR funded HIV-free program for comprehensive HIV interventions, including the elimination of mother-to-child transmission (eMTCT) of HIV and policy for routine antenatal HIV testing.

### Sampling strategy and participant selection

We employed two sampling techniques to recruit study participants. Purposive sampling was first used to recruit pregnant or postpartum participants based on pre-specified criteria like demographic characteristics (age, marital status, profession and educational status) and HIV testing status (a mix of couples who tested for HIV during pregnancy and those who did not test). Theoretical sampling was used to further recruit participants based on emerging concepts or to further saturate categories under development [36].

We advertised our study verbally during group antenatal and infant welfare clinics. Interested participants were provided with information sheets detailing the study objectives and contact information of the researchers. All male partners in attendance were approached for consent while a subgroup of women were approached for the phone number of their male partners who were not in attendance.

### Data collection

Data collection took place between July and December 2021 in English or Pidgin (Cameroonian creole) through in-person, semi-structured interviews (SSIs) and focus group discussions (FGDs). Open-ended SSIs and FGDs guides (Supporting information 1) with probes were developed based on the literature and our prior experience with male partner HIV testing [25, 37]. Topics included: sources of maternal HIV infection, maternal HIV risk perception, perspectives on couple communication and male partner testing.

Both group discussions and semi-structured interviews were conducted in tandem to allow for conceptual iteration and triangulation [38]. Specifically, concepts that emerged in one method were further explored or confirmed in its counterpart method [38, 39]. For example, after conducting the first 21 interviews, preliminary analysis was conducted and this was used to refine and explore concepts in group discussions. Emerging concepts in group discussions were similarly explored in subsequent interviews allowing for constant comparisons. All group discussions took place in a private room within the health facility while structured interviews either took place in the facility or community.

Data collection was led by the primary researcher (LHF) and a team of trained research assistants—a Nurse Midwife (NMN) assisted in conducting semi-structured interviews and a Sociologist (MT) who observed group discussions and took notes. SSIs sessions lasted between 22 and 56 mins while and FGDs sessions lasted between 74 to 120 mins. Participants received snacks and transport reimbursement where applicable. Debriefing sessions during data collection were held between three authors (LHF, HS and NMN) to review preliminary concepts and the researcher’s reflective diary (analytic memo). This informed subsequent participant sampling and clarified conceptual categories. In total, 93 potential participants were approached and 80 agreed to participate—36 men (*n* = 30 SSIs; *n* = 6, FGD) and 44 women (*n* = 38 SSIs; *n* = 6, FGD).

### Data analysis

Interviews and group discussions were audiotaped with participant permission and transcribed verbatim. In keeping with the Constructivist Grounded Theory approach, data collection and analysis occurred iteratively and concurrently—that is concepts that emerged during initial interviews and group discussions informed subsequent sampling, data collection, analysis and vice versa [33].

Through memoing and constant comparison, we coded data in three phases: initial, focused and theoretical coding [34, 36]. At the initial phase, two team members (LHF and NMN) read transcripts line-by-line and generated over 75 codes and memos while ensuring that they were grounded in participants’ data [33]. These codes were discussed with the broader team—further examined for connections and compared for commonalities and variations through focused coding—resulting in initial codes being clustered into 15 focused categories [33, 34]. This was finalised with theoretical coding and hybrid saturation (data set and theory) [40] whereby we integrated focused categories into a core category that was underpinned by maternal HIV risk perception, testing outcome and moderated by couple communication, proxy testing and facility engagement. We further saturated this with additional interviews resulting in the construction of a grounded theory on the mechanism and social processes that influence male partner testing behaviours during pregnancy [33, 41]. This approach distilled and transitioned our focused codes from description to conceptualisation [33]. Nvivo Windows (Release 1) © Qualitative Research Solutions International (QSR) [42] and Microsoft programmes were used to manage and analyse data.

## Results

The demographic characteristics of study participants is shown in Table 1. The median age was 30 years for women and 31 years for men. Almost all were in a couple (married or cohabiting). Fifty-two percent of women and 75% of men had formal education beyond primary level. Only seven women (16%) stated that they were not in any form of employment while all men reported that they were involved in income-generating activities. Regarding reports about male testing, only 14% of female respondents said that their male partner had tested for HIV but 25% of male respondents said they had been tested.

**Table 1:** Participant demographics

Characteristic	Pregnant/postpartum couple Participants	
	**Female** ***N* = 44**	**Male** ***N* = 36**
**Age (median)**	30	31
**Relationship status**		
Married (%)	33 (75)	25 (69)
Cohabiting (%)	9 (21)	11 (31)
Single(%)	2 (4)	0 (0)
**Completed secondary or high school education**		
Yes (%)	23(52)	27(75)
No (%)	21(48)	9(25)
**Employed**		
Yes (%)	37 (84)	36 (100)
No (%)	7 (16)	0(0)
**Reported male partner HIV testing**		
Yes (%)	6 (14)	9(25)
No (%)	38 (86)	27(75)

Our analysis revealed that male partner testing is shaped by five intersecting themes: (1) perceived maternal HIV risk, (2) couple HIV communication, (3) perceived outcome of spousal testing, (4) proxy testing, (5) limited male partner engagement by health staff. These concepts are further expanded and interpreted with supporting quotes from participant narratives below.

### A. Perceived maternal HIV risk

The core category centred on participants’ perceptions of maternal HIV risk. Perception was mixed and largely grounded in assumptions around the sexual behaviours of pregnant women and their male partner during pregnancy.

#### Pregnant women are stable and men don’t approach them

The presumed fidelity of pregnant women and the normative perception that links HIV infection to promiscuity largely contributed to how maternal HIV acquisition was perceived. According to a majority of participants, pregnancy had a protective effect on maternal risk behaviours. Pregnant women were presumed to be stable and faithful compared to non-pregnant women who were labelled ‘loose and free’—with a high likelihood of risky sexual behaviours and HIV acquisition.

*“A pregnant woman is steady and does not have time for outings compared to a non-pregnant woman who is free to flirt around. So a non-pregnant woman is more at risk of contracting HIV”—30yr old male participant*

#### Pregnant women are abandoned sexual projects— men go out and bring HIV

In the context of male partner behaviour, participants centred their perception on maternal HIV risk on the perceived physiological changes that make pregnant women less appealing and hesitant to respond to sexual advances from their partners. Consequently, pregnant women were labelled ‘abandoned sexual projects’ by male partners who explored extramarital sex to satisfy their needs—thereby predisposing their pregnant partners to HIV.

*“…It is generally known that a woman is like an abandoned sexual project from pregnancy through childbirth and may be about 2-4months after birth… Coupled with the fact that women don’t give ‘it’ on time [hesitate to have sex], and we have needs as men. Some men may decide to go out there and bring the virus to their pregnant wives. So pregnancy increases a woman’s chance of contracting HIV” —35 & 21 yr old Male FGD Participants*

*“When women are pregnant, their partners seize the opportunity to ‘go out’(have affairs) and bring HIV. This increases a woman’s risk of having HIV” —34yr old female participant*

### B. Perceived outcome of spousal testing

Participants revealed that the outcome of spousal testing was an underlying factor that determined male partner involvement in antenatal testing. This was defined in terms of perceived benefits and consequences or risk associated with partner testing. The need to confirm or reinforce fidelity, for example through safer sexual practices, awareness for timely action, fear of discordance and consequently abandonment or relational dissolution were frequently cited outcomes that influence partner testing. Sub-categories that emerged in this thematic area are expanded and supported with participant quotes below:

#### Testing together confirms fidelity

Participants associated HIV acquisition with either promiscuity or extramarital affairs. A partner’s acceptance to test was therefore motivated by the need to confirm one’s fidelity and reinforce spousal respect.

*When couples test together, it is a sign of fidelity. It shows they have nothing to hide. It strengthens relationships in the sense that it gives the partner a level of confidence that I am faithful because if I was not sure of myself, I wouldn’t have accepted to come” —41yr old male participant*

*In my case, when we went through the HIV test during pregnancy our joy was different. Even my husband now respects me.—27yr old female participant.*

*If you don’t know your status, you don’t really know anything*

Irrespective of perceived testing outcome, participants reported that testing together is motivated by the need for awareness which could lead to timely intervention and a deterrent to extramarital affairs. Among female participants, a concordant negative test result was particularly perceived as an incentive for male partners to renew their commitment to marital fidelity.

*“Coming to test is a first step because if you don’t know your status, you don’t really know anything— If it is negative, it clears the air. If it is positive, it is positive and the couple can take better actions”.*

*—27yr old, female participant*

*“Joining us to test might stop men’s fidgeting and cheating. If there were things he was doing prior to the test, once he discovers that he is at least free, he might be afraid to go out again because something bad might happen” —39yr old, female participant*

*The relationship will get burned if the results don’t match*

#### The relationship will get burned if the results don’t match

The possibility of a sero-discordant HIV test result emerged as a common determinant of male partners not engaging in HIV testing. This was mostly perceived as a deterrent to couple testing and disclosure. Respondents cited instances where community reports and personal experiences of HIV discordancy led to the dissolution of relationships.

*“I have the case of a friend. I don’t know how it happened, the wife tested positive, and the man at that time was negative. They went for the test together, but came back separately and it has since not been well with them” —41yr old, male participant*

*“Some partners are hesitant to come and test together because the relationship would not only be broken… it will get burned if the results don’t match” —39yr old, male participant*

The consequences of discordance was especially severe among female participants who did not only report the potential loss of intimacy but added that for women who mostly depend on male partners for economic sustenance, disclosure of a positive test result led to the loss of their source of livelihood.

*“When I came and did my test and it turned out positive, my husband was just aloof; he did not want to be intimate with me. When I requested something, he did not want to provide” —25yr old, female participant*

#### Without the knowledge of HIV, they were living happily and blindly

The lack of acceptance in the event of a positive test result was cited as a common phenomenon among discordant couples. The bliss of blind ignorance was therefore preferred over the potential turmoil an awareness of a partner’s status might bring.

*Testing as a couple comes with a risk because most times when one is positive, it is very difficult to make the other partner accept the positive partner meanwhile without the knowledge of HIV, they were living happily and blindly—36yr old, female participant*

### C. Couple communication

#### Without the knowledge of HIV, they were living happily and blindly

Related to maternal HIV risk perception, spousal communication on HIV emerged as a cross-cutting theme that drives male partner testing. Several participants reported that communication was rare and when it did occur, the novelty of the relationship or suspicion for infidelity was the stimulus for communicating a request for HIV testing.

*“We used to communicate on HIV more at the beginning of our relationship when we were dating. Not anymore. I don’t know why, but maybe we trust ourselves enough”—30yr old, female participant*

Additionally, couple communication was said to be mostly initiated by women who used indirect, subtle and suggestive approaches to either bring up safe sexual practices or insist on partner testing.

*“We don’t discuss HIV directly. She once said: ‘you travel a lot. Get a condom into your bag because anything can happen’. I wondered why she did not come directly to caution me against going after another woman’ ” —33yr old, male participant*

### D. Proxy testing

The misconception that a woman’s HIV test result reflects her partners’ result was a recurrent theme in our study. This was especially compounded by presumed partner fidelity and disclosure of a negative test result by pregnant partners who are mandated to test during their first antenatal visit.

#### You have tested for the two of us—we are one

Participants reported that male HIV risk perception was relatively low especially when a pregnant partner has previously tested and disclosed a negative test result. With the claim of spousal ‘oneness’, men therefore sent their pregnant partners to test in order to gain a proxy indication of their own status.

*“Men wonder how they can first of all have HIV. They say, first of all go and test, when you test, you have tested for the two of us. We are one”26yr old, female participant*

*“If my wife tested negative, automatically mine will be negative also…It has not been long since I did my HIV testing and she just did hers and it was negative so, it means I’m also negative”—39yr old, male participant*

*I am also testing for my husband*

#### I am also testing for my husband

In the context of presumed partner fidelity, risk perception and claims on proxy testing were mixed. Some pregnant women declined the need for partner testing due to the perceived absence of HIV risk—suspected infidelity or a sexually transmitted infection. In the event of suspected infidelity, women used their test results as an approximation of their partner’s status.

*“The past couple of years I have been testing regularly and since he has never given me any issues to think that he is cheating on me and I have not had any funny STDs apart from yeast cell, it just makes me think he is ‘clean’ and does not need to come for the test”—34yr old, female participant*

*“Generally, when a woman starts asking the husband for an HIV test, it means someone is being suspected. As a result, a woman might decide to remain silent or go for her test saying ‘as I am doing this test, I am also testing for my husband’ ” —39yr old, male participant*

#### He too should be testing

A subset of female participants however rejected the notion of gauging their partner’s status by proxy testing. They therefore expressed the need for partner testing because they could not fully vouch for the fidelity of their male partners.

*“If I am testing, he too should be testing. I cannot consider my own test as my husband or fiancé’s own…[sigh] you know the nature of our men nowadays, they cheat… he will need to do it”— 31 & 34 years old female participants*

### Limited male partner engagement by health staff

#### No one asked him to do the test

The limited engagement of male partners for HIV testing by facility staff also emerged as a theme. Despite attending antenatal clinics as a couple, some participants reported that providers did not initiate HIV counselling and testing for their male partners.

*“...no one asked. He was just there with me, we moved together from one stage of the antenatal consultation to the next but no one asked him to do the test”—25yr old, female male participant*

## Discussion

**Fig. 1:**
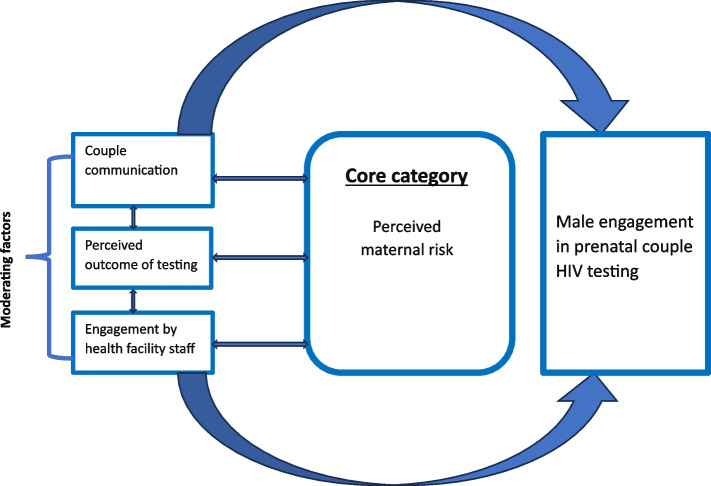
A grounded theory on factors that influence male partner engagement in prenatal HIV testing in Cameroon

Our study sought to expand understanding on the factors that influence male involvement in antenatal HIV testing. As seen on Fig. 1 below, our analysis of participant perspectives offers a novel grounded theory that identifies maternal HIV risk perception as a prominent theme (core category) that intersects with perception on HIV testing outcome, couple communication and engagement of male partner by facility staff to influence couple prenatal HIV testing. This finding expands previous studies that offered inconclusive evidence on how these factors intertwine and compound to influence couple HIV testing in Cameroon and sub-Saharan Africa [24, 25, 43, 44].

Our data uncovered the interplay between maternal HIV risk perception and the motivation for male partner testing. As a primary factor, it overlapped with the presumed behaviours of pregnant women and their male partners to influence the decision to engage or not engage in couple antenatal testing. Across all participant groups, respondents reported that pregnancy offered a protective effect on maternal HIV acquisition because pregnant women were perceived as stable and less likely to engage in risky sexual behaviours compared to their non-pregnant counterparts. With such views, most men thought they were less at risk of contracting HIV from their pregnant partners and therefore saw no need to engage in antenatal HIV testing. Paradoxically, contrary perspectives emerged when maternal HIV risk depended on the behaviours of male partners. While female pregnancy status was perceived to be a buffer for both women and their partners, this was not the case when the perception on male behaviours during pregnancy was taken into account. We also found that other mediating factors not directly linked to actual HIV risk undermined the uptake of couple testing, including a previously negative maternal HIV test result (proxy testing), absence of a sexually transmitted infection and estimated confidence in a partner’s faithfulness. This finding corroborates previous research on the psycho-social and cultural framing of HIV risk perception and the uptake of HIV prevention strategies [45–47]. It also feeds into the backdrop of the gendered dimension of HIV and sexual behaviours where the moral agency on HIV prevention is unfairly situated on the behaviours of women who are also expected to be HIV testing representatives for male partners despite the high prevalence of extramarital affairs in the latter [16–18, 48, 49]. Thus, improving male engagement in antenatal HIV testing context will require counselling and behaviour change communication strategies that challenge inaccurate risk perceptions, deconstruct negative masculine sexual ideals and situate the dual responsibility of HIV prevention equally on pregnant women and their male partners.

Regarding the acceptability of male partner testing, multilevel perceptions on the benefits and consequences of couple testing was shown to be a driver of couple testing. Previous research has shown the fear of testing positive to be a deterrent to couple testing [23, 28]. In our study however, fears extended beyond a positive test result to the possibility of HIV sero-discordancy which were reported to bring about relational dissolution and the loss of livelihood especially among female participants. Among male participants, the decision to test or not test was informed by the need to confirm fidelity, build trust and promote risk-reduction behaviours. This finding might be underpinned by HIV related stigma and the normative perception that it is solely transmitted through a sexual route, thereby casting doubts on the moral credibility of the HIV positive partner [50]. Hence, the need to upscale and deploy HIV prevention intervention aimed at limiting stigma and fears associated with being tested for HIV.

In our study, we found that couple communication was rare, indirect and subtle because bringing up the subject of HIV testing implied there was distrust or suspected infidelity. However, the possibility of an extra-marital affair or perceived maternal risk prompted female participants to indirectly suggest risk-reduction behaviours or the uptake of male-partner testing. This is in agreement with previous research suggesting that interspousal communication on HIV is a key determinant of male partner testing and is associated with risk-reduction behaviours [44, 51]. Additionally, while earlier literature on HIV is gendered and portrays men as dominant determinants of women’s HIV risk [15, 16], our findings present a nuanced understanding on the potential shifts in female assertiveness and autonomy regarding their sexual and reproductive health.

Our study had a number of limitations: it relied on self-reported data from participants, which can be influenced by social desirability bias or recall bias. Participants might underreport or over-report certain behaviors or perceptions. Additionally, the interpretivist paradigm and constructivist grounded theory approach rely heavily on the researchers’ interpretations, which can introduce subjectivity and potential bias in data analysis and theme identification. This was however mitigated by the diverse nature of our participant base and methodological triangulation of study findings. Also, the acceptability of couple testing were explored hypothetically. Future research on the feasibility and lived experience of HIV testing is needed to establish real-world evidence on how couples navigate HIV testing decisions in the antenatal context. Additionally, the perspectives of men and extent of sexual relationships outside their primary union will need to be explored.

## Conclusion

Our study explored the combined perspectives of pregnant women and their male partners and generated a novel model that expands understanding on the drivers of male partner HIV testing in the prenatal context. Importantly, we identified maternal HIV risk perception compounded by the intersection of couple communication and possibility of HIV testing outcome influence the decision to engage in or decline couple HIV testing. Finally, in order to bridge the gap in the first 95% of UNAIDS 95–95-95 goals, we propose two areas where interventions and policies could support male engagement in prenatal HIV testing: (1) couple education on accurate HIV risk perception including gender transformative approaches in HIV risk-reduction behaviours; (2) strategies to improve couple communication. Particularly, further research is needed to establish how the shift in gender dynamics and female empowerment may influence couple communication and shared decision-making on the uptake of HIV testing during pregnancy.

## Supplementary Information

Supplementary Material 1.

## Data Availability

All relevant data used to reach the conclusions drawn in the study are within the paper. This data has also been safely stored at the CBCHS- https://cbchealthservices.org/ and LSHTM- https://www.lshtm.ac.uk/. For reasons of confidentiality, original transcripts and demographic information is not available to the public as some of the interviewees are easily identifiable. This data could however be made available upon reasonable request.

## References

[1] UNAIDS. Global AIDS Update 2022. 2022. https://www.unaids.org/sites/default/files/media_asset/2022-global-aids-update-summary_en.pdf.

[2] Dunkle KL, Greenberg L, Lanterman A, Stephenson R, Allen S. Source of new infections in generalised HIV epidemics – Authors’ reply. The Lancet. 2008;372(9646):1300–1. 10.1016/S0140-6736(08)61547-0. 10.1016/S0140-6736(08)61545-7 18929899

[3] Dunkle KL, Stephenson R, Karita E, et al. New Heterosexually Transmitted HIV Infections in Married or Cohabiting Couples in Urban Zambia and Rwanda: An Analysis of Survey and Clinical Data. Vol 371. 2008. 10.1016/S0140-6736(08)60953-8. 10.1016/S0140-6736(08)60953-8 18586173

[4] Chemaitelly H, Awad SF, Shelton JD, Abu-Raddad LJ. Sources of HIV incidence among stable couples in sub-Saharan Africa. Published online. 2014. 10.7448/IAS.17.1.18765. 10.7448/IAS.17.1.18765 PMC3935448 24560339

[5] UNAIDS. Blind Spot - Reaching out to Men and Boys - Addressing the Blindspot in Response to HIV.; 2017. https://www.unaids.org/sites/default/files/media_asset/blind_spot_en.pdf

[6] Nardell MF, Adeoti O, Peters C, et al. Men missing from the HIV care continuum in sub-Saharan Africa: a meta-analysis and meta-synthesis. J Int AIDS Soc. 2022;25(3):e25889. 10.1002/JIA2.25889. 35324089 10.1002/jia2.25889 PMC8944222

[7] UNAIDS. Fast-Track Commitments To End AIDS by 2030.; 2016. https://www.unaids.org/sites/default/files/media_asset/fast-track-commitments_en.pdf

[8] WHO. Consolidated Guidelines on HIV Testing Services. 2015. https://www.who.int/publications/i/item/9789241508926.

[9] WHO. Guidance on Couples HIV Testing and Counselling Including Antiretroviral Therapy for Treatment and Prevention in Serodiscordant Couples. 2012. https://iris.who.int/handle/10665/44646. 23700649

[10] Slogrove AL, Powis KM, Johnson LF, Stover J, Mahy M. Estimates of the global population of children who are HIV-exposed and uninfected, 2000–18: a modelling study. Lancet Glob Health. 2020;8(1):e67–75. 10.1016/S2214-109X(19)30448-6. 31791800 10.1016/S2214-109X(19)30448-6 PMC6981259

[11] The World Bank. Population, total - Cameroon. World Bank Data. 2022. Accessed April 10, 2024. https://data.worldbank.org/indicator/SP.POP.TOTL?locations=CM

[12] UNAIDS. UNAIDS Data 2022.; 2022. Accessed April 10, 2024. https://www.unaids.org/en/resources/documents/2023/2022_unaids_data

[13] PEPFAR. CAMEROON Country Operational Plan (COP/ROP) 2019 Strategic Direction Summary. 2019. https://www.state.gov/wp-content/uploads/2019/09/Cameroon_COP19-Strategic-Directional-Summary_public.pdf.

[14] Minsante (Ministry of Public Health). Cameroon Population-Based HIV Impact Assessment (CAMPHIA) 2017–2018: Final Report. .; 2020. Accessed February 25, 2022. https://phia.icap.columbia.edu/wp-content/uploads/2021/09/53059-CAMPHIA-Report_EN_WEB_August1.pdf

[15] Dhillon S, Amoak D, Chidimbah Munthali GN, Sano Y, Antabe R, Luginaah I. Polygamy and safe sex negotiation among married women: evidence from Cameroon. BMC Infect Dis. 2023;23(1):1–12. 10.1186/S12879-023-08826-4/TABLES/4. 37993765 10.1186/s12879-023-08826-4 PMC10664310

[16] Bandali S. Norms and practices within marriage which shape gender roles, HIV/AIDS risk and risk reduction strategies in Cabo Delgado. Mozambique AIDS Care. 2011;23(9):1171–6. 10.1080/09540121.2011.554529. 21476146 10.1080/09540121.2011.554529

[17] Siu GE, Wight D, Seeley JA. Masculinity, social context and HIV testing: An ethnographic study of men in Busia district, rural eastern Uganda. BMC Public Health. 2014;14(1):1–11. 10.1186/1471-2458-14-33/PEER-REVIEW. 24417763 10.1186/1471-2458-14-33 PMC3893584

[18] Khumalo S, Taylor M, Makusha T, Mabaso M. Intersectionality of cultural norms and sexual behaviours: a qualitative study of young Black male students at a university in KwaZulu-Natal. South Africa Reprod Health. 2020;17(1):1–10. 10.1186/S12978-020-01041-3/TABLES/1. 10.1186/s12978-020-01041-3 PMC7687826 33234147

[19] Farquhar C, Kiarie JN, Richardson BA, et al. Antenatal Couple Counseling Increases Uptake of Interventions to Prevent HIV-1 Transmission NIH Public Access. 2004. 10.1097/00126334-200412150-00016. 10.1097/00126334-200412150-00016 PMC3384734 15577420

[20] Rosenberg NE, Graybill LA, Wesevich A, et al. The Impact of Couple HIV Testing and Counseling on Consistent Condom Use among Pregnant Women and Their Male Partners: An Observational Study. J Acquir Immune Defic Syndr (1988). Published online 2017. 10.1097/QAI.0000000000001398 10.1097/QAI.0000000000001398 PMC5493523 28426440

[21] NACC. PMTCT Progress Report 2017. http://www.cnls.cm/sites/default/files/rapport_progres_ptme_ndeg_12_2017_1.pdf

[22] Nannozi V, Wobudeya E, Gahagan J. Fear of an HIV positive test result: an exploration of the low uptake of couples HIV counselling and testing (CHCT) in a rural setting in Mukono district, Uganda. 10.1177/1757975916635079. 2016;24(4):33–42. 10.1177/1757975916635079 10.1177/1757975916635079 27235411

[23] Katz DA, Kiarie JN, John-Stewart GC, Richardson BA, John FN, Farquhar C. Male perspectives on incorporating men into antenatal HIV counseling and testing. PLoS One Published online. 2009. 10.1371/journal.pone.0007602. 10.1371/journal.pone.0007602 PMC2765726 19881884

[24] Nkuoh GN, Meyer DJ, Tih PM, Nkfusai J. Barriers to Men’s Participation in Antenatal and Prevention of Mother-to-Child HIV Transmission Care in Cameroon, Africa. J Midwifery Womens Health Published online. 2010. 10.1016/j.jmwh.2010.02.009. 10.1016/j.jmwh.2010.02.009 20630363

[25] Morfaw F, Mbuagbaw L, Thabane L, et al. Male involvement in prevention programs of mother to child transmission of HIV: a systematic review to identify barriers and facilitators. Syst Rev Published online. 2013. 10.1186/2046-4053-2-5. 10.1186/2046-4053-2-5 PMC3599633 23320454

[26] Byamugisha R, Tumwine JK, Semiyaga N, Tylleskär T. Determinants of male involvement in the prevention of mother-to-child transmission of HIV programme in Eastern Uganda: A cross-sectional survey. Reprod Health Published online. 2010. 10.1186/1742-4755-7-12. 10.1186/1742-4755-7-12 PMC2913932 20573250

[27] Mohlala BKF, Gregson S, Boily MC. Barriers to involvement of men in ANC and VCT in Khayelitsha, South Africa. In: AIDS Care - Psychological and Socio-Medical Aspects of AIDS/HIV. ; 2012. 10.1080/09540121.2012.668166 10.1080/09540121.2012.668166 PMC3613944 22519913

[28] Manjate Cuco RM, Munguambe K, Osman NB, Degomme O, Temmerman M, Sidat MM. Male partners’ involvement in prevention of mother-to-child HIV transmission in sub-Saharan Africa: A systematic review. SAHARA-J: Journal of Social Aspects of HIV/AIDS. 2015;12(1):87–105. 10.1080/17290376.2015.1123643 10.1080/17290376.2015.1123643 26726756

[29] Farquhar C, Osoti A, Han H, Kinuthia J. Role of male partners in the prevention of mother-to-child HIV transmission. Res Rep Neonatol. Published online July 2014:131. 10.2147/RRN.S46238

[30] WHO. Male Involvement in the Prevention of Mother-to-Child Transmission of HIV. World Health Organization; 2014. https://iris.who.int/bitstream/handle/10665/70917/9789241503679_eng.pdf.

[31] Takah NF, Kennedy ITR, Johnman C. The impact of approaches in improving male partner involvement in the prevention of mother-to-child transmission of HIV on the uptake of maternal antiretroviral therapy among HIV-seropositive pregnant women in sub-Saharan Africa: A systematic review and meta-analysis. BMJ Open Published online. 2017. 10.1136/bmjopen-2017-018207. 10.1136/bmjopen-2017-018207 PMC5719335 29175889

[32] Foglabenchi LH, Stöckl H, Marchant T. ‘I am a father but not pregnant’: a qualitative analysis of the perspectives of pregnant couples on male partner role during pregnancy care in Bamenda Cameroon Abstract Reproductive Health. 2024;21(1). 10.1186/s12978-024-01928-5. 10.1186/s12978-024-01928-5 PMC11665113 39716286

[33] Charmaz K. Constructing Grounded Theory. 2nd ed. SAGE Publications Ltd; 2014. Accessed May 31, 2024. https://books.google.co.uk/books/about/Constructing_Grounded_Theory.html?id=v_GGAwAAQBAJ&redir_esc=y

[34] Charmaz K. Constructivist grounded theory. Journal of Positive Psychology. 2017;12(3):299–300. 10.1080/17439760.2016.1262612.

[35] Cameroon Baptist Convention Health Services. Nkwen Baptist Health Center-Overview. 2019. Accessed February 25, 2022. https://cbchealthservices.org/health-centers/north-west-region/nkwen-baptist-hc/

[36] Glaser, B.G., & Strauss A. The Discovery of Grounded Theory: Strategies for Qualitative Research. Aldine; 1967.

[37] Ditekemena J, Koole O, Engmann C, et al. Determinants of male involvement in maternal and child health services in sub-Saharan Africa: A review. Reprod Health. 2012;9(1):1–8. 10.1186/1742-4755-9-32/FIGURES/1. 23171709 10.1186/1742-4755-9-32 PMC3573948

[38] Lambert SD, Loiselle CG. Combining individual interviews and focus groups to enhance data richness. J Adv Nurs. 2008;62(2):228–37. 10.1111/J.1365-2648.2007.04559.X. 18394035 10.1111/j.1365-2648.2007.04559.x

[39] Lewis J, Ritchie J, Ormston R, Morrell G. Generalising from qualitative research. In: J. Ritchie JLCMN& RO, ed. Qualitative Research Practice : A Guide for Social Science Students and Researchers. 2nd ed. SAGE Publications Ltd; 2014:xxiv, 430 pages : illustrations ; 24 cm.

[40] Drisko JW. Strengthening Qualitative Studies and Reports. J Soc Work Educ. 1997;33(1):185–97. 10.1080/10437797.1997.10778862.

[41] Varpio L, Ajjawi R, Monrouxe LV, O’Brien BC, Rees CE. Shedding the cobra effect: problematising thematic emergence, triangulation, saturation and member checking. Med Educ. 2017;51(1):40–50. 10.1111/MEDU.13124. 27981658 10.1111/medu.13124

[42] QSR International. NVivo Windows (Release 1). Published online 2020. Accessed May 31, 2024. https://help-nv.qsrinternational.com/20/win/Content/welcome.htm#

[43] Musoke P, Hatcher A, Rogers AJ, et al. Men’s hopes, fears and challenges in engagement in perinatal health and the prevention of mother-to-child transmission of HIV in rural Kenya. Cult Health Sex. 2018;20(11):1259–72. 10.1080/13691058.2018.1426785. 29465291 10.1080/13691058.2018.1426785 PMC6103893

[44] Orne-Gliemann J, Tchendjou PT, Miric M, et al. Couple-oriented prenatal HIV counseling for HIV primary prevention: An acceptability study. BMC Public Health Published online. 2010. 10.1186/1471-2458-10-197. 10.1186/1471-2458-10-197 PMC2873579 20403152

[45] Brewer NT, Weinstein ND, Cuite CL, Herrington JE. Risk perceptions and their relation to risk behavior. Ann Behav Med. 2004;27(2):125–30. 10.1207/S15324796ABM2702_7. 15026296 10.1207/s15324796abm2702_7

[46] Sjöberg L. Explaining risk perception: an empirical evaluation of cultural theory1. Risk Management. Published online June 18, 2020:127–144. 10.4324/9780429284243-8

[47] Maughan-Brown B, Venkataramani AS. Accuracy and determinants of perceived HIV risk among young women in South Africa. BMC Public Health. 2017;18(1):1–9. 10.1186/S12889-017-4593-0/TABLES/3. 28732496 10.1186/s12889-017-4593-0 PMC5520344

[48] Uwah C, Wright S, Uwah C, Wright S. Socio-Cultural Identities, Perceptions of Sexuality/Sexual Behavior and Cultural Contexts as Determinants of HIV and AIDS Prevalence in Southern Africa. World J AIDS. 2012;2(1):17–23. 10.4236/WJA.2012.21003.

[49] Patel RC, Leddy AM, Odoyo J, et al. What motivates serodiscordant couples to prevent HIV transmission within their relationships: findings from a PrEP implementation study in Kenya. Cult Health Sex. 2018;20(6):625–39. 10.1080/13691058.2017.1367421. 28903628 10.1080/13691058.2017.1367421 PMC5851810

[50] Larsson EC, Thorson A, Nsabagasani X, Namusoko S, Popenoe R, Ekström AM. Mistrust in marriage-Reasons why men do not accept couple HIV testing during antenatal care - A qualitative study in eastern Uganda. BMC Public Health. 2010;10(1):1–9. 10.1186/1471-2458-10-769/TABLES/1. 21167040 10.1186/1471-2458-10-769 PMC3018443

[51] Musoke P, Darbes L, Hatcher AM, et al. Couple Efficacy and Communal Coping for HIV Prevention Among Kenyan Pregnant Couples. AIDS Behav. 2022;26(7):2135–47. 10.1007/S10461-021-03559-4. 35122576 10.1007/s10461-021-03559-4 PMC9167231

