# Supplementary material for: ‘They went for the test together but came back separately’: a constructivist grounded theory perspective on male engagement in antenatal HIV testing in Bamenda, Cameroon
Source: BMC Pregnancy Childbirth. 2025 Jan 22;25:57. doi: 10.1186/s12884-025-07134-w (PMC11756143; doi:10.1186/s12884-025-07134-w)
Supplement: Supplementary file 1 — Supplementary Material 1. [file 12884_2025_7134_MOESM1_ESM.docx]

**TOPIC GUIDES for Semi-Structured Interviews and Focus Group Discussions**

**Male partner role and engagement in pregnancy care and HIV prevention: pregnant couples and health worker perspectives in Cameroon**

**OUTLINE**

1. Introduction about the study (purpose, procedure, anticipated benefits, reporting of findings, informed consent, permission to record etc)
2. Rapport Building: Mainly demographic information
3. Interview proper
4. Closing of interview (thanking the participant; reminding them about commitments for confidentiality and privacy from research team)
5. **Perceptions and behavior norms on male partner involvement in antenatal care and HIV-prevention during pregnancy**

**Semi-Structured Interviews**

**Couple prenatal HIV testing & pattern of communication**

- What services do you know that are offered in antenatal care? What are the HIV related services?
- What do you know about HIV transmission during pregnancy? (perceived risk of transmission/acquisition during pregnancy)
- How do you feel about being asked to attend antenatal care visits with your partner to receive HIV testing? Would you prefer receiving HIV advice alone or with your partner? Why?
- What are some of the barriers to receiving HIV testing as a couple?
- What are some benefits of receiving HIV testing as a couple? (early treatment, prevent transmission)
- Do you think attending antenatal care and testing together for HIV with your partner will affect your relationship? If yes, what impact do you think it would have on your relationship? If no, why do you think this would not have any effect on your relationship?

**HIV related Communication (or lack thereof) with partner regarding Prenatal HIV testing)**

- Have you ever had a discussion on HIV with your partner? If no, why? If yes, who starts the discussion? What do you mainly talk about?
- Have you discussed HIV-testing with your partner during this pregnancy? How did it go?

**For those who received testing with their partner: Please tell me about your experience receiving HIV testing during prenatal care with your partner**

***Possible probing topics:***

- How did the staff treat you?
- How long did you have to wait to be seen?
- Did you have to pay for anything because of your visit? How much?
- Did you receive appropriate counseling prior to testing as well as afterwards?
- How did your partner react to you receiving testing? (appreciative versus tension?)
- Did the experience result in any positive outcomes? Any negative outcomes?
- Would you do it again?

**Focus Group Discussions**

- What do you know about HIV transmission during pregnancy? (perceived risk of transmission/acquisition during pregnancy)
- What are some of the barriers to receiving HIV testing as a couple?
- What are some benefits of receiving HIV testing as a couple? (early treatment, prevent transmission)
- Do you think attending antenatal care and testing together for HIV will affect marital relationships? If yes, what impact do you think it would have on relationships? If no, why do you think this would not have any effect on relationships?
